# High fructose corn syrup ınduced liver and heart damage are not reversed with hazelnut consumption: In vivo study

**DOI:** 10.1371/journal.pone.0336329

**Published:** 2025-11-07

**Authors:** Ayça Toprak-Semiz, Efsane Yavuz-Bedir, Hakan Yüzüak, Murat Usta, Demet Şengül

**Affiliations:** 1 Department of Medical Pharmacology, Faculty of Medicine, Recep Tayyip Erdoğan University, Rize, Türkiye; 2 Department of Medical Services and Techniques, Vocational School of Health Services, Giresun University, Giresun, Türkiye; 3 Department of Physiology, Faculty of Medicine, Giresun University, Giresun, Türkiye; 4 Department of Medical Biochemistry, Faculty of Medicine, Giresun University, Giresun, Türkiye; 5 Department of Pathology, Faculty of Medicine, Giresun University, Giresun, Türkiye; The Islamic University, IRAQ

## Abstract

Hazelnut, antioxidant, anti-inflammatory effects, has an important role in a healthy diet. High fructose corn syrup (HFCS), used as a sweetener in ready-made food, beverages; causes hyperlipidemia, fatty liver, cardiovascular system damages; oxidative stress, inflammation play role in these damages. Based on these data, we aimed to examine liver and heart damage caused by HFCS in rats and to investigate possible role of hazelnut enriched food in preventing/improving these damages. During this process, weight change, food, liquid consumption were recorded. Biochemical parameters were measured with standard enzymatic techniques. Inflammatory cytokines were determined by ELISA. Liver and heart tissues were evaluated histopathologically, changes were scored, graded. HFCS decreased food, increased liquid consumption. Feeding with hazelnut reduced fluid consumption. HFCS increased weight gain, hazelnut did not reverse it. LDH, CK values increased in HFCS group due to heart damage. While damage occurred in livers of HFCS group due to increased levels of TNF-α and IL-1ß, feeding with hazelnut did not change it. In heart, inflammatory cytokines were similar between groups. In histopathological analysis, inflammation was observed both in livers, hearts of HFCS group. In hazelnut group, a significant decrease in damage was observed compared to HFCS, HFCS+H groups. According to our results, hazelnut supplementation reduced liquid intake and showed limited cardiac protection, but did not reverse HFCS-induced hepatic or cardiac injury.

## Introduction

Hazelnut is an essential component of the Mediterranean diet, a dietary model frequently cited as a reference for healthy nutrition [1]. Hazelnuts is rich in mono- and polyunsaturated fatty acids, plant proteins, fiber, vitamins, phytosterols, and polyphenolic antioxidants. It is known to modulate lipid metabolism, reduce systemic inflammation, oxidative stress and improve reproductive system health. Through these mechanisms, hazelnut has been associated with cardiovascular and reproductive system protection and a reduction in diet-related metabolic disorders [2–6].

In contrast, high-fructose corn syrup (HFCS), a sweetener widely used in processed foods and beverages, has repeatedly been linked to adverse health effects in both pre-clinical and clinical studies. HFCS promotes hepatic lipogenesis, disrupts insulin signaling, and increases pro-inflammatory cytokine release, thereby contributing to hepatic steatosis, hyperlipidemia, insulin resistance, and cardiovascular injury [7–9]. The growing consumption of HFCS-containing products, coupled with modern dietary habits, has made these health risks a global concern.

The detrimental effects of HFCS are closely linked to inflammatory responses and structural alterations at the tissue level, which can be evaluated through biochemical parameters, inflammatory markers, and histopathological scoring. Although oxidative stress is also implicated in HFCS-induced damage, our study specifically focused on inflammatory markers, biochemical parameters, and histopathological scoring to directly capture the organ-specific consequences of HFCS and the potential protective effects of hazelnut supplementation. This design allows for a more targeted evaluation of the mechanisms driving hepatic and cardiac injury.

Based on these data, we aimed to investigate the liver and heart damage caused by HFCS in rats and the role of hazelnut in preventing or healing the damages. Although previous research has separately highlighted the health-promoting effects of hazelnuts and the detrimental outcomes of HFCS, to our knowledge, no study has yet examined their interaction in a controlled experimental model. Our study is the first to examine the relationship between the possible therapeutic effects of hazelnut feeding on the harmful effects of HFCS for liver and heart. The novelty of this study lies in addressing this gap by simultaneously assessing biochemical, inflammatory, and histopathological outcomes in the liver and heart. By integrating these complementary measures, the study provides new evidence for the therapeutic potential of hazelnuts against diet-induced metabolic and cardiovascular damage.

## Materials and methods

### Animals, ethics statement and diet

All procedures involving animals were in compliance with the European Community Council Directive of 24 November 1986, and ethical approval was granted by Giresun University Local Ethics Committee (Ethic no: 2021/08). In this study, only male Wistar albino rats were used to minimize variability caused by estrous cycle–related hormonal fluctuations, as estrogen and progesterone can influence metabolic and inflammatory responses. Previous work has also shown sex-related differences in response to HFCS, with estrogen suggested to provide partial protection against weight gain in females [10]. Besides, their use is consistent with most HFCS-related models reported in the literature [11–13]. Thirty-two male, 9 weeks aged, weighing 100–150 g Wistar albino rats were maintained at a constant temperature of 20–22ºC, with a humidity of, 60 ± 5%, 12 hour of light and 12 hour of darkness. During the entire experiment period (10 weeks), the animals were housed in Giresun University Animal Research Laboratory, under the supervision of a veterinarian, standard rat pellet feed and hazelnut feed were used for feeding ad libitum. A 10-week duration was chosen based on prior studies where cardiac/hepatic metabolic and histological changes induced by HFCS were consistently observed [11–14]. After acclimation for two weeks, the rats were divided into 4 research groups: control (C); high fructose corn syrup (HFCS), hazelnut treated HFCS (HFCS+H) and hazelnut (H), each group consisting of 8 rats. Fluid consumption (water or HFCS) was also ad libitum. At the end of ten weeks, the rats were sacrified by exsanguination (bloodless) method during surgery. In order to perform the surgical procedure, the animals were first deeply anesthetized by intraperitoneally (ip) injecting the combination of the active Ketamine (80 mg/kg, Ketasol®) and Xylazine (10 mg/kg, Xylazinbio®). During 10 week, a rat from control group died. After sacrification, blood samples were collected from the heart for biochemical analysis. Half of the liver and heart tissues were placed into falcon tubes and stored at −80°C for biochemical analyses and another half of the tissues were fixed in falcon tubes with 10% formaline solution for histopathological analyses.

### Preparation and administration of HFCS and hazelnut

HFCS was prepared as 30% (weight/volume) from a concentrated solution (containing 42% fructose, 53% glucose, provided as a gift from a beverage company in Giresun) freshly and daily as drinking water to the animals in HFCS and HFCS+H groups for 10 weeks. HFCS concentrations were set according to the sugar content of many soft drinks in which sugar content from 7% to 15% [11,15,16].

The hazelnuts used in the experiments were obtained from Fiskobirlik^®^ store in Giresun, as raw hazelnut kernel with skin. The hazelnuts provided were sent to a special feed producing company (Arden Research & Experiment) and 5.5% hazelnuts were added to the standard feed content by this company, suitable for the experimental animals, and turned into pellet feed. Hazelnut feeds were produced isocalorically (equivalent in calories) with control feed in order not to cause differences in consumption amounts. The animals were fed with a standard rodent chow diet that composed of 53,63% starch, 22% protein, 11,6% fat, 6,39% cellulose and 6,38% ash and standart vitamins. Hazelnut-supplemented feed corresponded to ~3 g/kg/day per rat (≈0.9 g/rat/day), equivalent to ~5.5% of the total diet. This calculation was based on the typical food intake of adult male Wistar rats (15–25 g/day) [17] and their higher metabolic rate compared with humans [18]. The chosen dose corresponds to ~0.4 g/kg/day or ~30 g/day for humans, consistent with Mediterranean diet recommendations [19,20]. The same dietary formulation has been applied in previous hazelnut-supplemented rodent studies [1,4,5] supporting translational relevance and comparability. Liquid and food consumption were measured daily, and body weights were recorded weekly.

### Biochemical analyses

Blood samples taken from the heart were collected into tubes and centrifuged at 4°C, 10000 g for 30 minutes. After centrifugation, serum Troponin T (hsTnT), Alanine Aminotransferase (ALT), Aspartate Aminotransferase (AST), Creatine Kinase (CK), Lactate dehydrogenase (LDH), glucose, total cholesterol, triglyceride, HDL-Cholesterol, LDL-Cholesterol, Uric acid levels were determined by standard enzymatic techniques; analyzed with Cobas® 8000 modular (Roche Diagnostics GmbH, Mannheim, Germany) auto-analyzer by using commercial kits at Medical Biochemistry Laboratory, Giresun Training and Research Hospital, Giresun University.

All biochemical parameters were reported with appropriate units: ALT, AST, CK, and LDH in U/L; Troponin T in ng/L; glucose and lipid parameters (total cholesterol, triglyceride, HDL, LDL) in mg/dL; uric acid in mg/dL. Values are expressed as mean ± SEM or median (25th–75th percentile).

### Histopathological analyses

Liver and heart tissues were fixed with 10% formalin, processed through graded alcohols and xylene, and embedded in paraffin blocks. Sections (4–6 μm) were stained with hematoxylin and eosin (H&E) according to standard pathology laboratory procedures (Giresun Training and Research Hospital, Giresun University) and examined under light microscopy (Nikon® ECLIPSE Ci–L) by a pathologist. Values are expressed as median (25th–75th percentile). Histopathological changes were scored using a semi-quantitative system (0 = no structural damage, 1 = minimal, 2 = moderate, 3 = severe) as described previously [9,10].

### ELISA

Liver and heart tissues isolated from the rats were stored at −80°C until the day of analysis. For protein extraction, the tissues were homogenized with lysis buffer (Lysis Buffer 2, Cloud-Clone Corp.) containing 1 mL of 1–2 mM PMSF (Cloud-Clone Corp.) on ice. After homogenization process, the samples were centrifuged at 10000xg for 5 minutes at 4°C and analyzes were performed on the supernatant. Tissue protein levels were determined by the turbidimetric method using the benzethonium chloride method using commercial kits (Roche Diagnostics GmbH) and expressed as mg/dL. The experimental protocol was carried out according to the instructions of the manufacturer of ELISA kits (Wuhan USCN Business Catalog No: SEA133Ra, SEA563Ra, SEA079Ra). The absorbance values of the samples were measured at 450 nm wavelength with the help of a microplate reader (Accureader, Metertech®). After establishing the equation of the graph obtained with the absorbance and concentration values of the standards, the concentrations of the samples were calculated by using the equation [10]. To account for variations in protein yield, cytokine concentrations obtained from ELISA were normalized to the corresponding total protein content of each sample. Final results were expressed as ng/g tissue.

### Statistical analyses

Statistical analyses were performed using Graphpad 5.0 and MedCalc® Statistical Software version 22.001 (MedCalc Software Ltd, Ostend, Belgium). Results for descriptive statistics, continuous variables with normal distribution were presented as mean ± standard error, n was the number of rats. Sample size was estimated a priori using G*Power 3.1.9.7. Based on a one-way ANOVA design with four independent groups (n = 8 per group), assuming an effect size of f = 3.07, α = 0.05, and desired power = 0.95, the required sample size was calculated as 8. With 32 rats in total, our study exceeded this requirement and achieved an actual power of 0.9995.

Continuous variables and ordinal variables with non-normal distribution were shown as median (25^th^ percentile – 75^th^ percentile). In comparisons of more than two groups, one-way ANOVA analysis, for post-hoc comparisons Tukey’s/Dunn tests were used for variables with normal distribution. In comparisons of more than two groups, Kruskal-Wallis H test was used for variables that were not normally distributed and Dunn test was used for post-hoc comparisons. For comparisons of expected and observed frequencies, Pearson chi-square test was used. When p value was less than 0.05 considered as statistically significant.

## Results and discussion

### The effects of HFCS and hazelnut on food, liquid intake and body weight

The amount of food consumption in HFCS, HFCS+H and Hazelnut groups decreased significantly versus control group. Food consumption amounts of HFCS and HFCS+H groups were similar. The amount of food consumption in HFCS and HFCS+H groups were also significantly different versus Hazelnut group (Table 1).

**Table 1:** Effects of HFCS and hazelnut on food intake, liquid intake and body weights in groups of rats.

	Control	HFCS	HFCS+H	Hazelnut
**Food Intake (g/day)**	21,34 ± 0,30	8,80 ± 0,19 ^**a,b**^	8,31 ± 0,13 ^**a,b**^	20,27 ± 0,20 ^**a**^
**Liquid Intake (ml/day)**	52,40 ± 0,67	85,09 ± 1,33 ^**a,b**^	80,23 ± 1,36 ^**a,b,c**^	47,39 ± 0,67 ^**a**^
**Initial Body Weight (g)**	237,1 ± 8,48	226,6 ± 9,06	235,8 ± 8,18	228 ± 7,59
**Terminal Body Weight (g)**	307,8 ± 10,11	317,7 ± 13,29	331,3 ± 11,11	291 ± 7,99
**% Weight Change**	29,98 ± 2,33	40,15 ± 1,46 ^**b,d**^	40,68 ± 2,63 ^**b,d**^	27,92 ± 1,96

Values were expressed as mean ±SEM, n = 7–8. ^**a**^: p < 0,05 HFCS, HFCS+H, Hazelnut vs Control;

^**b**^: p < 0,05 HFCS, HFCS+H vs Hazelnut ^**c**^: p < 0,05 HFCS+H vs. HFCS ^**d**^: p < 0,05 HFCS, HFCS+H vs Control.

When the amount of liquid consumption was compared between the groups, significant increments were observed at HFCS and HFCS+H groups versus control and hazelnut group. The amount of liquid consumption of hazelnut group decreased compared to control. Similarly, liquid consumption of HFCS+H group was significantly lower than HFCS group (Table 1).

The initial and final weights of the experimental animals were compared between groups. According to the results, there was no significant difference between their initial and final weights (Table 1). When compared in terms of weight change and % weight change, it was observed that there was no difference between control and hazelnut groups and between HFCS and HFCS+H, but the weight gain in HFCS and HFCS+H groups were significantly higher versus control and hazelnut groups (Table 1).

### The effects of HFCS and hazelnut on biochemical parameters

ALT median values were significantly lower in HFCS+H group compared to control group. Median values of CK and LDH were significantly higher in HFCS group vs control group. Triglyceride median values were significantly lower in hazelnut group compared to the HFCS+H and HFCS groups. HDL-cholesterol median values were significantly lower in hazelnut group compared to HFCS+H and HFCS groups (Table 2).

**Table 2:** Effects of HFCS and hazelnut on biochemical parameters in groups of rats.

Biochemical Parameters	Control	HFCS	HFCS+H	Hazelnut	p
**hsTnT (µg/L)**	2,16 ± 0,45	5,04 ± 1,21	4,19 ± 1,1	3,46 ± 0,95	0,252
**ALT (IU/L)**	49 (46-51)	32 (25-39)	24 (19–32) ^**a**^	35 (31-39)	**0,008**
**AST (IU/L)**	99,0 ± 10,33	134,1 ± 22,24	123,5 ± 27,49	113,9 ± 13,7	0,669
**CK (IU/L)**	486 (417-712)	1705 (834–4545) ^**b**^	750 (508-1557)	750 (679-904)	**0,031**
**LDH (IU/L)**	571,0 ± 100,4	972,6 ± 118,8 ^**b**^	909,8 ± 175,2	598,5 ± 59,32	**0,032**
**Glucose (mg/dL)**	266,0 ± 11,17	337,4 ± 16,04	320,5 ± 17,82	286,3 ± 28,2	0,082
**Total Cholesterol (mg/dL)**	45,4 ± 1,49	49,0 ± 2,36	48,9 ± 2,53	43,0 ± 1,78	0,146
**Triglyceride (mg/dL)**	53 (38-70)	103 (71-173)	104 (60-112)	37 (31–58) ^**c,d**^	**0,005**
**HDL- Cholesterol (mg/dL)**	22,9 ± 1,1	28,6 ± 2,01	27,9 ± 1,63	21,6 ± 0,82 ^**c,d**^	**0,003**
**LDL- Cholesterol (mg/dL)**	7,86 ± 0,6	8,29 ± 0,94	8,00 ± 0,53	8,38 ± 0,53	0,939
**Uric acid (mg/dL)**	0,63 (0,52−0,94)	0,97 (0,90−1,14)	0,73 (0,58−1,08)	0,73 (0,54−0,89)	0,127

Values were expressed as mean ±SEM or median (25^th^ percentile-75^th^ percentile), n = 7–8. ^**a**^: p < 0,05 HFCS+H vs. Control; ^**b**^: p < 0,05 HFCS vs. Control; ^c^: p < 0,05 Hazelnut vs. HFCS+H; ^d^: p < 0,05 Hazelnut vs. HFCS.

### The effects of HFCS and hazelnut on liver and heart histology

**Fig 1:**
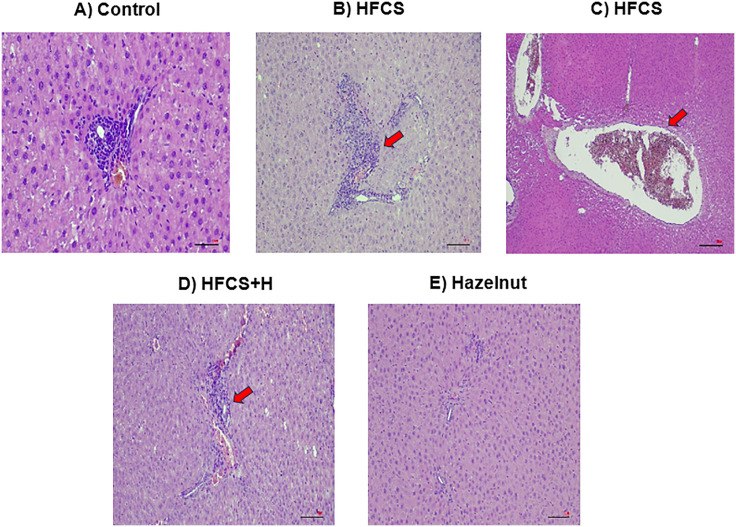
Histopathological appearance of the liver. **(A)** Control: normal histological profile, **(B)** HFCS group: inflammation (red arrow), **(C)** HFCS group: vascular congestion (red arrow), **(D)** HFCS+H group: inflammation (red arrow), **(E)** Hazelnut: normal histological profile were observed; n = 7-8.

According to histopathology results; inflammation, vascular congestion were observed in the liver of HFCS group, inflammation was not recovered in HFCS+H group, control group had also mild inflammation and hazelnut group showed normal profile (Fig 1).

**Fig 2:**
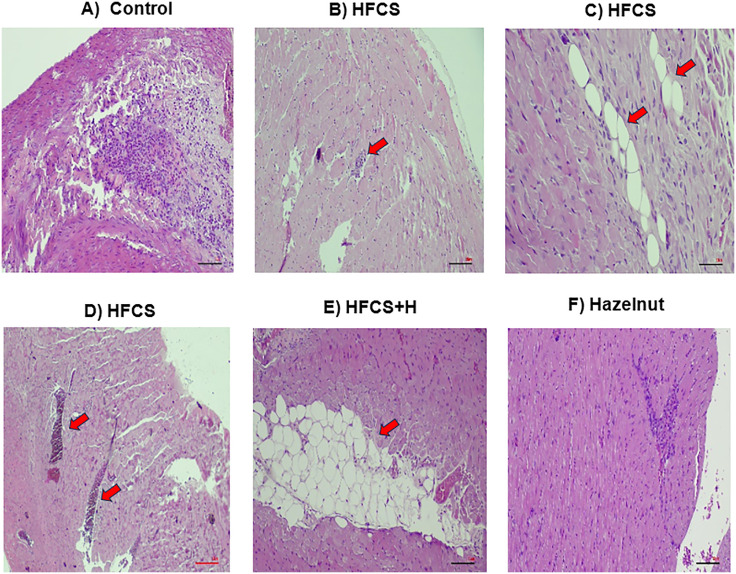
Histopathological appearance of the heart. **(A)** Control: normal histological profile, **(B)** HFCS group: inflammation (red arrow), **(C)** HFCS group: lipidosis (red arrows), **(D)** HFCS group: vascular congestion (red arrows), **(E)** HFCS+H: lipidosis (red arrow), **(F)** Hazelnut: normal histological profile were observed; n = 7-8.

Inflammation, vascular congestion and lipidosis were observed in the heart of HFCS group, damage was not recovered and lipidosis was shown in HFCS+H group, control group had mild inflammation and hazelnut group showed normal profile (Fig 2).

Liver and heart tissues score median values were significantly higher in HFCS and HFCS+H groups versus hazelnut group (Table 3 and 4).

**Table 3:** Comparison results for n (%) of damage degree scores for liver and heart tissues.

Tissue	Damage Score	Control	HFCS	HFCS+H	Hazelnut	p value
Liver	0, n (%)	2 (%28.6)	0	0	4 (%50.0)	0.050
	1, n (%)	4 (%57.1)	4 (%50.0)	4 (%50.0)	4 (%50.0)	
	2, n (%)	1 (%14.3)	4 (%50.0)	4 (%50.0)	0	
	3, n (%)	0	0	0	0	
Heart	0, n (%)	2 (%28.6)	0	0	6 (%75.0)	**0.008**
	1, n (%)	4 (%57.1)	4 (%50.0)	7 (%87.5)	2 (%25.0)	
	2, n (%)	1 (%14.3)	2 (%25.0)	1 (%12.5)	0	
	3, n (%)	0	2 (%25.0)	0	0	

n = 7–8, (Liver and heart tissues were scored using a semi-quantitative system 0 = no structural damage, 1 = minimal, 2 = moderate, 3 = severe as described previously [9,10]).

**Table 4:** Comparison results for median (25th percentile-75th percentile) damage degree scores for liver and heart tissues.

Tissue	Control	HFCS	HFCS+H	Hazelnut	p value
Liver Tissue Score	1 (0–1)	2 (1 - 2)	2 (1–2)	1 (0–1) ^**a,b**^	**0.0082**
Heart Tissue Score	1 (0–1)	2 (1–3)	1 (1–1)	0 (0–1) ^**a,b**^	**0.0019**

^**a**^: p < 0,05 Hazelnut vs. HFCS; ^**b**^: p < 0,05 Hazelnut vs. HFCS+H; n = 7–8. (Liver and heart tissues were scored using a semi-quantitative system 0 = no structural damage, 1 = minimal, 2 = moderate, 3 = severe as described previously [9,10]).

### The effects of HFCS and hazelnut on inflammatory cytokines TNF-ɑ, IL-1ß and IL-6

**Fig 3:**
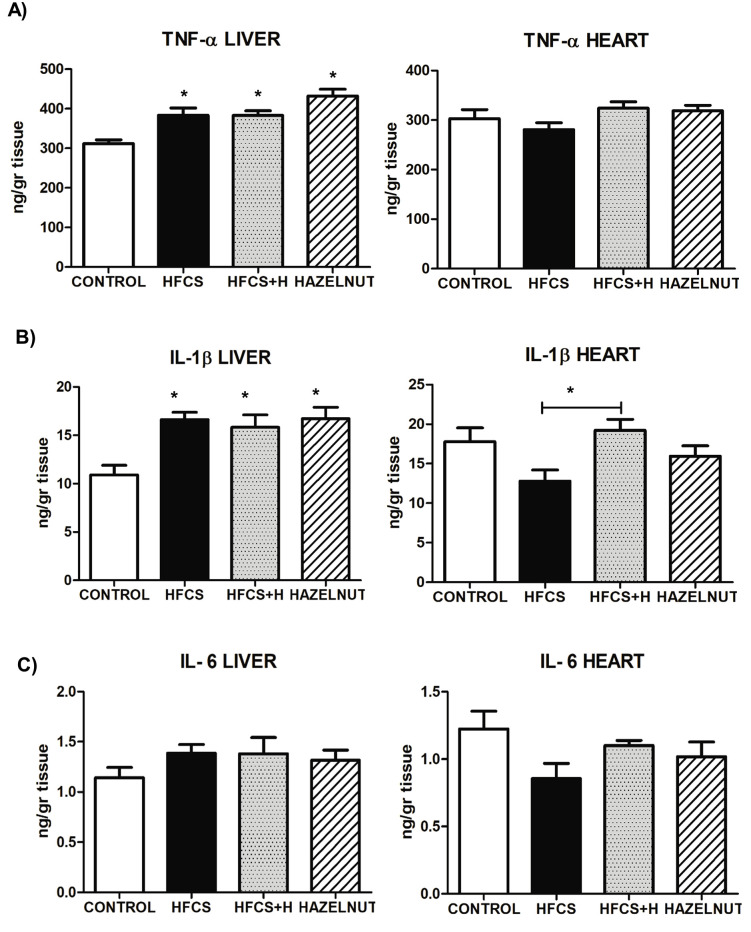
Measurement of inflammatory cytokines in liver and heart tissues. **A)** TNF-ɑ, **B)** IL-1ß, **C)** IL-6 levels, *: p < 0,05 HFCS, HFCS+H, Hazelnut groups vs. Control group for TNF-ɑ and IL-1ß levels of liver, Additionally, *: p < 0,05 HFCS+H group vs. HFCS group for IL-1ß levels of heart; Values were expressed as mean ±SEM, n = 7-8.

TNF-ɑ levels in the liver of HFCS, HFCS+H and hazelnut groups increased significantly compared to control group. But, TNF-ɑ levels in the heart did not change between the groups (Fig 3A).

When IL-1ß levels of liver were examined, significant increments were observed in HFCS, HFCS+H and hazelnut groups compared to control. Although there was a decrease in the HFCS+H group compared to the HFCS group, it was not statistically significant. For IL-1ß levels in the heart, there were not any differences in HFCS, HFCS+H and hazelnut groups versus control. Whereas, there was a significant increase in HFCS+H group compared to HFCS (Fig 3B).

IL-6 levels of the liver and heart did not differ between the groups (Fig 3C).

In our study, HFCS reduced food but increased liquid intake, consistent with reports of fructose-induced hyperdipsia [11,12,21]. Hazelnut supplementation did not alter food intake but decreased liquid intake, while a hazelnut-only diet reduced both food and liquid intake, suggesting potential effects on appetite and hydration. All groups gained weight from the fourth week, but percentage weight change was higher in HFCS groups, confirming its obesogenic effect. Hazelnut supplementation did not reverse this outcome, although a hazelnut-only diet tended to attenuate weight gain without significance. Prior studies are inconsistent, with some showing weight gain [14,22] and others reporting no differences [15,21,23,24] likely reflecting variation in design, duration, and dietary composition.

Blood glucose was elevated in the HFCS group compared with controls, and slightly lower in HFCS+H, but without significance. Similar nonsignificant results were reported in other HFCS studies [21,23,25] while some observed clear increases [24,26]. By contrast, hazelnut supplementation reduced glucose in high-fat diet models [27], suggesting that its glycemic effects depend on dietary background and metabolic context.

ALT and AST are standard markers of hepatic injury, yet in our study both showed only modest, non-significant changes. HFCS tended to increase AST and reduce ALT, while hazelnut supplementation had no clear effect. Previous HFCS studies reported inconsistent results, from decreases [13] to elevations [28]. Hazelnut oil, by contrast, lowered ALT and AST in a hamster fatty liver model [29], suggesting potential hepatoprotective effects of specific nut-derived compounds. Since AST is also expressed in cardiac and other extrahepatic tissues, and ALT is more specific to the liver, these enzymes should be interpreted together with histopathological and cytokine findings, which provided stronger evidence of HFCS-induced injury. The lack of a hazelnut effect may reflect the short intervention, modest dose, or use of whole kernels rather than extracts, limiting bioavailability.

HFCS consumption is known to disrupt lipid metabolism, yet in our study increases in cholesterol, triglyceride, HDL, and LDL were not significant. Triglycerides were significantly reduced in the hazelnut-only group compared with HFCS and HFCS+H, suggesting a selective lipid-lowering effect, whereas HDL levels also declined. Prior studies reported either nonsignificant lipid changes [7,25] or dyslipidemia with elevated cholesterol, triglyceride, and LDL [24,30]. Hazelnut-derived compounds such as filbertone have shown hypolipidemic effects in high-fat diet models [24] and hazelnut oil improved lipid profiles in hamsters with fatty liver disease [29]. These findings indicate that while HFCS alters lipid metabolism, hazelnut supplementation may modulate specific fractions in a dose- and context-dependent manner.

CK and LDH were significantly elevated in HFCS rats, confirming cardiac injury, while uric acid and Troponin T remained unchanged. Hazelnut supplementation slightly lowered CK and LDH but without significance. Similar HFCS-induced increases in CK and LDH have been reported, with inconsistent findings for uric acid [9,21,24]. Troponin T increased in HFCS and decreased in HFCS+H, but not significantly, contrasting with marked fructose-induced elevations in other studies [31]. Despite histological evidence of cardiac damage, serum Troponin T did not reach significance, likely due to short exposure duration or low-grade injury. Consistent with this, troponin elevations have been shown to remain mild and nonsignificant when cardiac injury is less severe [32].

Histopathological analysis showed significant cardiac and borderline hepatic alterations. Hazelnut supplementation lowered scores in both organs, but effects were modest and inconsistent. Microscopy confirmed HFCS-induced inflammation, vascular congestion, and cardiac steatosis. Similar liver damage has been reported [14], though some studies found no differences [21] and HFCS has been linked to cardiac congestion, lipid accumulation, and myocyte degeneration [9]. Hazelnut derivatives, such as leaf extract, demonstrated hepatoprotective effects in toxin-induced models [33]. Overall, HFCS induced structural damage in liver and heart, while hazelnut supplementation offered only limited protection.

Cytokine analyses showed hepatic increases in TNF-α and IL-1β across all groups, confirming HFCS-induced inflammation, while IL-6 remained unchanged. Hazelnut supplementation did not attenuate these effects, and elevations in the hazelnut-only group suggest context-dependent pro-inflammatory actions. In the heart, cytokines were largely stable, with only IL-1β elevated in HFCS+H. The unchanged IL-6 response may reflect its dual pro- and anti-inflammatory roles, or suppression under chronic dietary stress such as ER stress and hepatic IL-6 resistance [34,35]. Prior HFCS studies reported variable cytokine patterns, with increases [10,22,30] decreases in IL-6 [26], or no changes [7]. By contrast, hazelnut bioactives reduced cytokine expression in macrophages [36] and suppressed TNF-α and IL-6 in high-fat diet models [37]. Overall, these findings underscore the complex, context-dependent interplay between HFCS, inflammation, and nut-derived bioactives.

This study has several limitations. Only male rats were used, preventing assessment of sex-related effects. The 10-week intervention may have been too short to capture long-term outcomes, molecular/oxidative stress markers (e.g., MDA, SOD, GSH) and bioactive components were not measured, limiting mechanistic insight. Although statistical power was high (actual power >0.99), some findings such as liver histopathology reached only borderline significance, suggesting that hazelnut’s protective effects were modest. Despite these limitations, this is the first study to examine the combined effects of HFCS and hazelnut consumption on hepatic and cardiac tissues, and we believe the results contribute meaningfully to the field.

## Conclusion

In summary, hazelnut supplementation reduced liquid intake and showed limited cardiac protection, but did not reverse HFCS-induced hepatic or cardiac injury. To our knowledge, this is the first study to evaluate the combined effects of HFCS and hazelnuts on both organs, providing a basis for future work on dose, duration, and preparation. These findings may guide not only experimental research but also for potential clinical applications and the development of nutritional recommendations and public health policies.
